# Ghrelin-mediated inhibition of the TSH-stimulated function of differentiated human thyrocytes *ex vivo*

**DOI:** 10.1371/journal.pone.0184992

**Published:** 2017-09-20

**Authors:** Maria Barington, Marianne Møller Brorson, Jacob Hofman-Bang, Åse Krogh Rasmussen, Birgitte Holst, Ulla Feldt-Rasmussen

**Affiliations:** 1 Department of Medical Endocrinology, Rigshospitalet, University Hospital Copenhagen, Copenhagen, Denmark; 2 Institute of Pharmacology, Department of Neuroscience and Pharmacology, University of Copenhagen, Copenhagen, Denmark; Hokkaido Daigaku, JAPAN

## Abstract

Ghrelin is a peptide hormone produced mainly in the gastrointestinal tract known to regulate several physiological functions including gut motility, adipose tissue accumulation and hunger sensation leading to increased bodyweight. Studies have found a correlation between the plasma levels of thyroid hormones and ghrelin, but an effect of ghrelin on the human thyroid has never been investigated even though ghrelin receptors are present in the thyroid. The present study shows a ghrelin-induced decrease in the thyroid-stimulating hormone (TSH)-induced production of thyroglobulin and mRNA expression of thyroperoxidase in a primary culture of human thyroid cells obtained from paranodular tissue. Accordingly, a trend was noted for an inhibition of TSH-stimulated expression of the sodium-iodine symporter and the TSH-receptor. Thus, this study suggests an effect of ghrelin on human thyrocytes and thereby emphasizes the relevance of examining whether ghrelin also influences the metabolic homeostasis through altered thyroid hormone production.

## Introduction

Ghrelin is a 28 amino acid peptide hormone released mainly from the gastric oxyntic glands that acts through a G-protein coupled receptor called the ghrelin receptor (GhrR) [1]. The receptor is expressed in many regions of the brain including the hypothalamus [2], the anterior pituitary gland [2] and the hippocampus [3]. Moreover, though to a much lesser extent than in the pituitary, the receptor is also expressed in several peripheral tissues [4]. The most well-studied area of ghrelin is its influence on metabolic homeostasis in an orexigenic and adipogenic direction due to its effect on the energy expenditure, appetite, adipocyte metabolism [5–9] and gastrointestinal motility [10, 11]. All these effects of ghrelin, and maybe more, are likely to play a role in body mass homeostasis. Thus the plasma ghrelin concentration correlates negatively with the body mass [12, 13], weight loss correlates with an increase in plasma ghrelin [14] and weight gain with a reduction [15]. The production of ghrelin is influenced by the caloric load, ingested macronutrients [16] and external food cues [17].

The thyroid gland plays a central role in regulation of metabolism, why it is important to understand any interaction between ghrelin and thyroid function. Most studies have most consistently found an inverse correlation in untreated hyper- and hypothyroid patients and in the euthyroid state [18–29]. However, other studies found opposite correlations, especially among patients with Hashimoto's thyroiditis [28, 30–32] and subclinical hypothyroidism [33]. These association studies, however, do not reveal any causality between ghrelin and thyroid hormones but suggest a possible interaction of ghrelin with the hypothalamus-pituitary-thyroid (HPT) axis. Several intervention studies have been performed [5, 34–40]. *In vivo* studies in rats show that ghrelin injection causes a decline in thyrotropin releasing hormone (TRH) and thyroid-stimulating hormone (TSH) [5, 34, 35] as well as a decline of triiodothyronine (T_3_) and thyroxine (T_4_) [39, 40]. Similar studies in humans confirm the inhibiting impact of ghrelin on the plasma concentration of TSH [36, 38]; one [36], in contrast to the aforementioned studies [39, 40], also showing an elevated T_4_ plasma concentration. However, other studies in humans showed no effect [37]. A few studies have been performed to look further into the effect of ghrelin on the HPT axis *in vitro*. These studies will be described below.

GhrR is present in the hypothalamus [2], where ghrelin stimulates the activity of neuropeptide Y (NPY) and agouti-related protein (AGRP)-synthesizing neurons [41]. Activation of these two types of neurons has been shown to inhibit the activity of TRH neurons [42–44], but direct inhibition of the activity of TRH neurons by ghrelin has not been examined. Furthermore, the opposite functioning hormone leptin reduced fasting-induced increases in NPY and AGRP mRNA and prevented fasting-induced reduction in pro-TRH mRNA levels in the hypothalamus leading to a decrease in circulating thyroid hormone levels [45]. A similar study has not yet been performed for ghrelin. Less is known about the relationship between ghrelin and the thyrotrophs of the pituitary. Ghrelin stimulates the somatotrophs to synthesize GH [46] and studies also show the presence of GhrR in the thyrotrophs [47]. The percentage of thyrotrophs expressing GhrR in the pituitary seems to increase when mice are calorie restricted [47]. If this translates to humans, it might indicate that the pituitary could be causally and directly involved in the correlation between ghrelin and thyroid hormones in plasma. The potential function of ghrelin on the human thyroid isolated from the rest of the HPT axis is unknown, whereas two studies have been performed in rat thyroid cell lines [48, 49]. These studies showed an enhanced proliferation of the thyrocytes [48] and a potentiation of the TSH-induced expression of thyroglobulin (Tg), thyroperoxidase (TPO) and the sodium iodide symporter (NIS) by ghrelin [49].

The hypothesis of this study was that some of the adipogenic effect of ghrelin could be due to an impact on the thyroid-influenced metabolic rate. Thus, the present study is the first to investigate a direct effect of ghrelin on the TSH-induced human thyroid cell function *ex vivo*.

## Material and methods

### Primary thyroid cell cultures

Tissue samples from 9 patients were obtained from thyroidectomies due to non-toxic thyroid adenomas performed at the Department of ENT-Head and Neck surgery, Rigshospitalet, University of Copenhagen. The study was performed with the participants’ written informed consent and approval by the Danish Committees on Health Research Ethics, Capital region (Protocol number: H-1-2012-110) which also represents the institutional review board in Denmark. Prior to the operation the patients had not received any drugs known to influence the function of the thyroid. The paraadenomatous thyroid tissue was washed in a calcium and magnesium free PBS (Gibco, Invitrogen Thermo Fischer Scientific, Waltham, MA, USA). The tissue samples were sliced before incubation with collagenase I (1 mg/mL) (Sigma-Aldrich, St. Louis, MO) and dispase II (2.4 mU/L) (Roche, Basel, Schwitzerland) at 37°C for 75 min. The suspension was filtered through a 100 μm pore strainer (Falcon, BD bioscience, NJ) and cultured in HAM’s F-12 medium supplemented with 1% L-glutamin (Panum Institute, Copenhagen University, Denmark), 1% non-essential amino acids (Gibco, Invitrogen, Carlsbad, CA, USA), 5% fetal bovine serum (FBS) (Biological Industries, Beit HaEmek, Israel), 1% penicillin and streptomycin (Invitrogen) which will be referred to as the *medium mixture* for the remainder of this article. It was then centrifuged at 1200 x G for 5 minutes and re-suspended in the above mentioned medium mixture after addition of six nutritional factors: TSH (1 IU/L) (Sigma-Aldrich, St. Louis, MO), insulin (10 mg/L) (Eli Lilly, Herlev, Denmark), transferrin (6 mg/L) and glycyl-histidyl-lysine acetat (10 μg/L) (Sigma-Aldrich, St. Louis, MO), somatostatin (10 μg/L) (Calbiochem, EMD Millipore, Billerica, MA) and hydrocortisone (10^−8^ M) (Calbiochem, EMD Millipore, Billerica, MA). The cells were cultured under similar conditions to the cell line of epithelium cells from rats (FRTL-5) [50]. The cells were cultured as monolayers in a humidified atmosphere (5% CO_2_) at 37°C until a confluent monolayer was visualized in the wells for a maximum of 10 days. Afterwards, cells were starved from TSH for 72 hours and the following measurements were carried out in the presence of ghrelin (10^−7^ M) (PolyPeptide, Limhamn, Sweden), the above mentioned 6 nutritional factors including varying concentrations of TSH (0.1; 0.5; or 1 IU/L) and in absence of FBS. In optimization experiments, ghrelin at 6 different concentrations from 10^−11^ to 10^−6^ M gave almost similar responses and 10^−7^ M was chosen arbitrarily in the remaining experiments. Cell cultures were exposed for 72 hours after which supernatants and cells were harvested. Relevant controls without TSH and/or ghrelin were included. Cell supernatants were temporarily stored at -20°C until used for cAMP and Tg measurements described in section 2.1.2 and 2.1.3, respectively. For real-time quantitative polymerase chain reaction (RT-qPCR) analysis, cell remnants from the cultures incubated with 0.1 IU/L TSH were harvested using incubation with lysis buffer (Qiagen, Hilden, Germany) followed by addition of 70% ethanol and the preparation was stored at -80°C until analysis. Tissue samples from two more patients were amplified by RT-qPCR before culture and after 1, 5, 12 days and 6, 9, 10, 13 days, respectively, to examine the stability of the expression of the GhrR-1a (henceforth referred to as GhrR) in the thyroid cells compared to human brain GhrR (Table 1). The amplification products were aligned with the glyceraldehyde-3-phosphate dehydrogenase (GAPDH) reference gene (Table 1).

**Table 1:** Primer sequences for RT-qPCR.

Gene	Forward primer (5’-3’ Sequence)	Reverse primer (5’-3’ Sequence)
Tg	GGGCGGGCAGTCAGCAGAGAGTG	ACCATAGTGGGCAGCCTCGGGTGAG
TSH-R	GAATGCTTTTCAGGGACTATGCAAT	ACAGCAGTGGCTTGGGTAAGAA
TPO	GGAGAGTGCTGGGATGGAAG	GGATTTGCCTGTGTTTGGAA
NIS	CCTTAGCTGACAGCTTCTATGCCA	CCCCAAGAAAAACAGACGATCC
IL-6	AGAGTAACATGTGTGAAAGCAGCAA	CCTCAAACTCCAAAAGACCAGTGA
GhrR-1a	ACCAGAACCACAAGCAAACC	CAGGCTCAAAGGATTTGGAA
GAPDH	CATGAGAAGTATGACAACAGCCT	AGTCCTTCCACGATACCAAAGT

Tg, Thyroglobulin; TSH-R, thyroid-stimulating hormone receptor; TPO, thyroperoxidase; NIS, sodium iodide symporter; IL-6, interleukin 6; GhrR-1a, growth hormone secretagogue receptor 1a; and GAPDH, glyceraldehyde-3-phosphate dehydrogenase.

#### cAMP

3-Isobutyl-1-methylxanthine (IBMX) diluted in alcohol (final ethanol concentration 1%) was used for cAMP assessment and added to the cell cultures concurrently with ghrelin. The negative controls were added 1.1% ethanol. Cells were harvested as described above, and the cAMP concentration was measured by a competitive protein binding method as described elsewhere [51] in which paper controls stimulating cells with forskolin were performed. The detection limit of the assay was 0.004 μM [52]. The calibration range was 0.05 to 2.0 μM. The intra-assay variation at the concentration of 0.4 μM was 4.7% and 7.2% at the concentration of 1.4 μM (n = 8 duplicates for each control level). For the low control, the inter-assay variation was 13.5% (range 0.29–0.45 μM) and 9.7% for the high control (range 1.10–1.71 μM) (n = 5 samples in duplicates for each control) [52].

#### Thyroglobulin

The Tg levels were assessed in supernatants by enzyme-linked immunosorbent assay (ELISA). Wells of polystyrene microtiter plates were coated with mouse anti-human Tg-antibody (Tg-Ab) (TF33, 3.1 g/L, AbD Serotec, Oxford, UK) and blocked with 200 μL TBS/0.5% bovine serum albumin (BSA) for 20–24 hours at 4°C. The plates were washed and incubated with supernatants for 60 minutes at 37°C and with rabbit anti-human Tg-Ab (K14, diluted 1:2 x 10^5^) for another 60 minutes at 37°C. After washing, peroxidase-conjugated polyclonal porcine anti-rabbit immunoglobulin (P399, Dako, Glostrup, Denmark, diluted 1:2 x 10^3^) and murine serum (Dako) were added and incubated for 60 minutes. Plates were washed again and a chromogenic substrate was added (TMB One, KEM EN TEC diagnostics, Taastrup, Denmark). Sulphuric acid (0.18 M) was added to stop the reaction and the results were measured by an ELISA reader (BioTek Synergy 2) at 450 nm. The calibration range was 10 to 500 μg/L [52]. When ELISA was performed, the samples were diluted until Tg levels measured were in compliance with the detection range. Afterwards, the results were adjusted in accordance with the dilution series. The intra-assay variation at the concentration of 52 μg/L was 9.5% and 8% at 101 μg/L (n = 7 and 6 duplicates for the low and high control level, respectively). For the low control, the inter-assay variation was 22.3% (range 32–65 μg/L) and 17.5% for the high control (65–121 μg/L) (n = 5 samples in duplicates for each control) [52].

#### RT-qPCR

RT-qPCR was used for measuring the mRNA encoding Tg, NIS, TPO, TSH receptor (TSH-R) and interleukin-6 (IL-6), the latter used for controlling the specificity of the ghrelin-induced effect. Total RNA was extracted from cultured, primary human thyroid cells from 7 patients with Qiagen Rneasy mini kit according to the manufacturer’s protocol. NanoDrop spectrophotometer was used for quantification of isolated RNA. For each sample, cDNA was synthetized (Superscript VILO synthesis kit, Invitrogen) by mixing 4 μl of the VILO reaction mix, 2 μl of the Superscript enzyme mix, the RNA (same amount from each sample) and RNAase free water to a total volume of 20 μl. Samples were incubated for 10 minutes at 25°C, 60 minutes at 50°C and 5 minutes at 85°C, whereupon 80 μl of 0.5X Tris-EDTA-buffer (Sigma-Aldrich) were added. The RT-qPCR analysis was performed with SYBR® Green JumpStart Taq Ready Mix (Sigma-Aldrich). A pool of undiluted cDNA was used for standards. 4 μl of SYBR Green JumpStart Taq ReadyMix, 10 μl of H_2_O and 1 μL of primers (1μM final concentration of each primer) were added to each reaction. RT-qPCR was performed on Lightcycler 480 II (Roche, Basel, Switzerland) with an initial denaturation at 94°C for 2 minutes, 45 cycles consisting of 30 seconds at 94°C, 45 seconds at 59°C, 1.30 minutes at 72°C. The analysis was followed by a melting curve analysis. The cycle threshold (Ct) values obtained from the RT-qPCR were normalized to the reference gene beta-2-microglobulin (Table 1).

### Statistics

Results were analyzed in GraphPad Prism 7 (2016 GraphPad Software, Inc.) and represented as means + or ± SEM. P-values lower than 0.05 were considered statistically significant. All experiments were carried out in triplicate. When cell cultures with and without ghrelin with the same concentration of added TSH were compared, the paired, the non-parametric Wilcoxon signed-rank test was used (i.e. in all statistics performed) [53, 54]. In the Tg and cAMP assays, the basal levels, i.e. the values in the absence of TSH, were subtracted, before the groups were compared. Absolute values for the basal levels of Tg and cAMP are shown in the *Supporting information* section.

## Results

### Ghrelin receptors are expressed in thyroid cells

**Fig 1:**
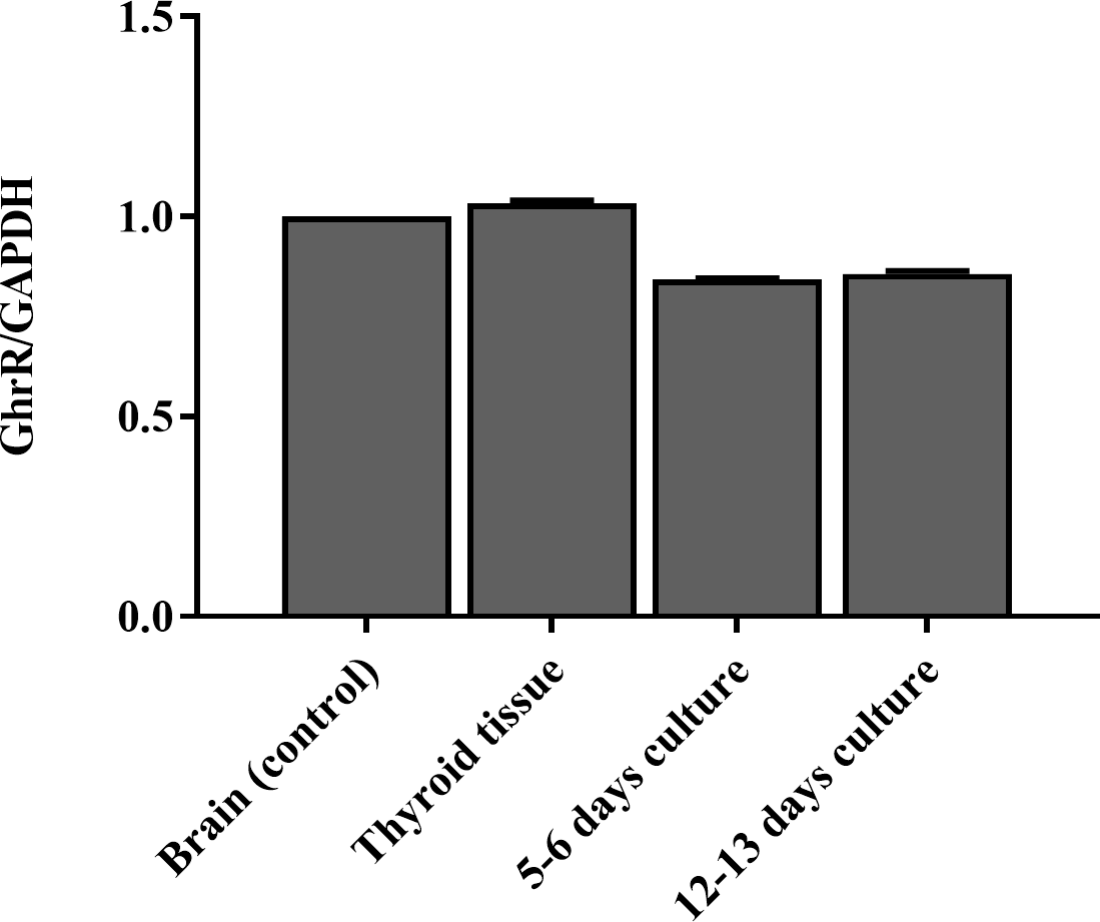
Ghrelin receptor (GhrR) mRNA expression level. GhrR mRNA expression level in relation to the reference gene glyceraldehyde-3-phosphate dehydrogenase (GAPDH) mRNA expression level in human brain, thyroid tissue and cell cultures measured by real-time quantitative polymerase chain reaction (RT-qPCR). n = 2.

To ensure that the culturing procedure of the thyroid tissue did not affect the expression level of GhrR we tested the expression of the receptor in two human thyroid tissue samples before culture and after 12–13 days of culture. We found that the concentration of GhrR in the thyroid was about the same as in human brain tissue before and after 12–13 days (Fig 1).

### Role of ghrelin as an inhibitor of TSH-induced thyroglobulin production

**Fig 2:**
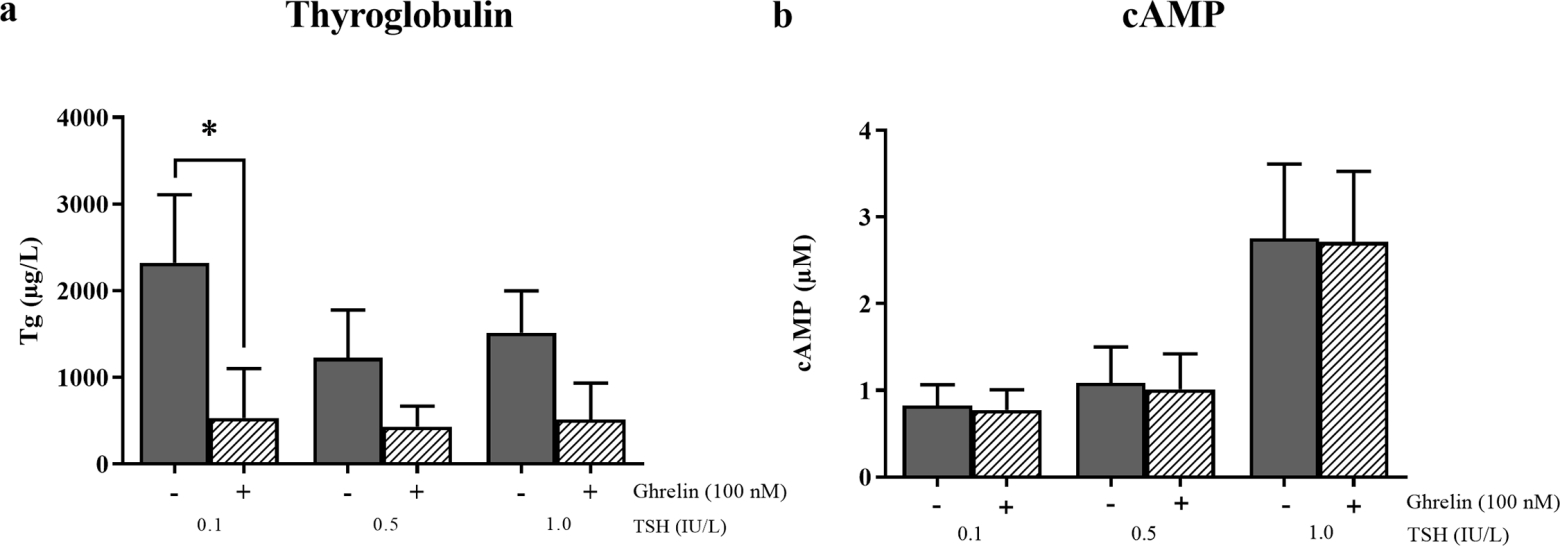
The influence of ghrelin on the thyroid-stimulating hormone (TSH)-induced increase in thyroglobulin (Tg) and cAMP production. The influence of ghrelin on the TSH-induced increase in Tg and cAMP production at three different concentrations of TSH (0.1 IU/L, 0.5 IU/L and 1 IU/L). The basal levels, i.e. the values in the absence of TSH, were subtracted, before the groups were compared. Grey = vehicle, pattern = ghrelin (100 nM). Means (+SEM). *P < 0.05 compared to the control (vehicle). **A)** Ghrelin inhibited the TSH-induced increase in Tg production measured by enzyme-linked immunosorbent assay (ELISA) in primary cultures of human thyroid cells for the TSH concentration of 0.1 IU/L. n = 8 (0.1 IU/L) and n = 6 (0.5 and 1 IU/L) in triplets. Two patient samples were excluded due to lack of basal TSH-induced Tg production. **B)** No influence of ghrelin on the TSH-induced increase in cAMP production at three different concentrations of TSH (0.1 IU/L, 0.5 IU/L and 1 IU/L) measured by a competitive protein binding method in primary cultures of human thyroid cells. n = 8 (0.1 IU/L and 1 IU/L) and n = 6 (0.5 IU/L) in triplets.

To test whether ghrelin affected the TSH-induced Tg production, we treated cells with TSH alone and in combination with ghrelin, respectively. TSH stimulated an equal increase in Tg production at all three concentrations used, in accordance with a former study [53] in which the TSH effect on Tg production was the same at 0.1 to 10 IU/L TSH. After addition of ghrelin, a significant decrease in the 0.1 IU/L TSH-induced production of Tg was observed (n = 8, p = 0.039) (Fig 2A). Although not significant, the same tendency was seen for concentrations of TSH of 0.5 and 1 IU/L in which a decrease was found (n = 6, p = 0.16 for both). Importantly, ghrelin only decreased the TSH-induced Tg production, whereas the production of Tg without TSH stimulation was unaffected by ghrelin treatment (S2A Fig).

### Ghrelin did not influence the TSH-stimulated cAMP generation

To see which part of the TSH receptor signaling pathway that ghrelin inhibits, we examined if ghrelin treatment affected TSH-induced cAMP expression. No effect of ghrelin on the TSH-induced production of cAMP was observed which indicates that the inhibiting effect on the ghrelin-induced Tg secretion is at least not involving the steps upstream of the adenylate cyclase in the G_αs_ coupled pathway of TSH (Fig 2B). It should be noted that some of the cAMP values measured are below calibration range, though above the detection limit. However, this does not change the conclusion that no effect of ghrelin on the TSH-induced production of cAMP was found.

### Ghrelin decreased the TSH-induced expression of TPO

**Fig 3:**
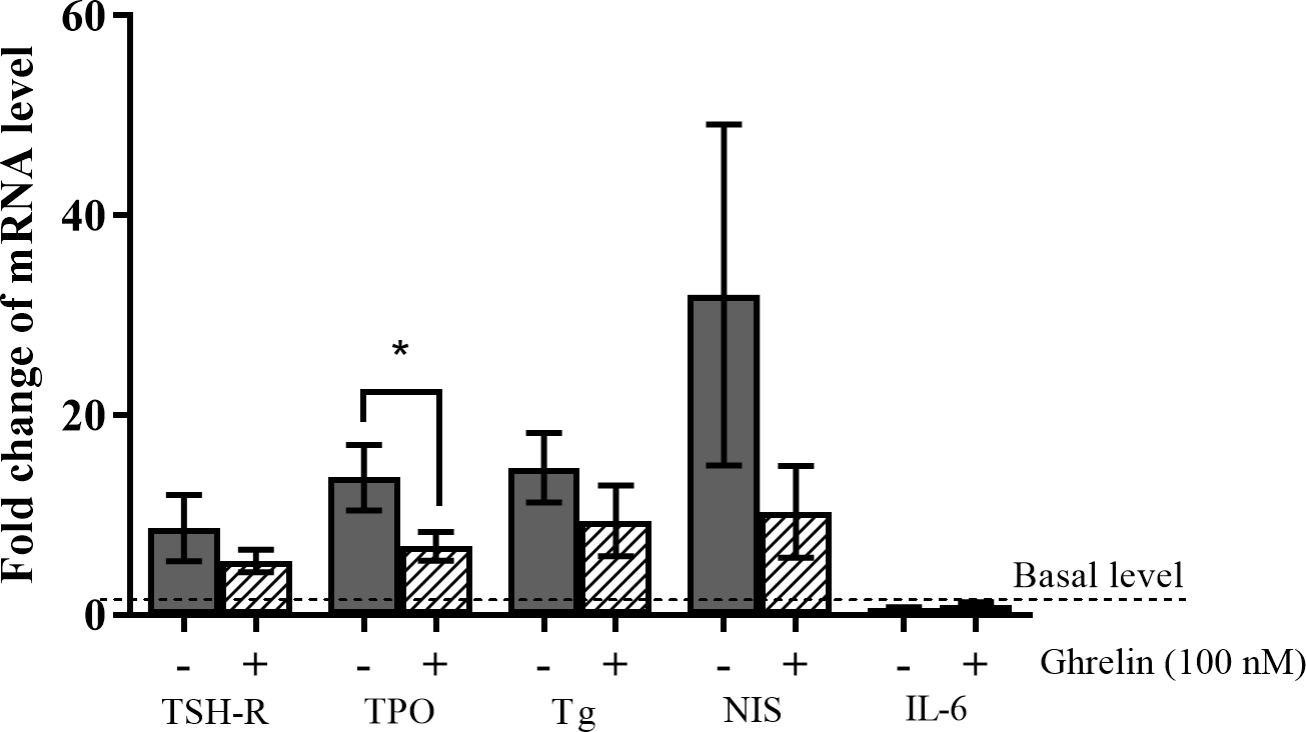
The influence of ghrelin on the thyroid-stimulating hormone (TSH)-induced (0.1 IU/L) mRNA expression of four thyroid components. The expression of the TSH receptor (TSH-R), thyroperoxidase (TPO), thyroglobulin (Tg) and sodium iodide symporter (NIS) measured by real-time quantitative polymerase chain reaction (RT-qPCR) in a primary culture of human thyroid cells in presence and absence of ghrelin. Indicated as fold change of mRNA expression compared to basal level (dashed line). IL-6 was used as a negative control. Grey = vehicle, pattern = ghrelin (100 nM). Means (±SEM), n = 6. *P < 0.05 compared to the control (vehicle). Two patients were excluded due to unknown sample material.

To analyze changes in key thyroid components upon the addition of ghrelin, we performed RT-qPCR analysis of the expression levels of Tg, NIS, TPO and TSH-R. All cell cultures responded to the addition of TSH (0.1 IU/L) by multi-fold increases above the basal levels (Fig 3). Combined addition of ghrelin and TSH inhibited TPO upregulation significantly (p = 0.031). Tg, NIS and TSH-R decreased as well, though not obtaining statistical significance. Importantly, the reference gene IL-6 was unaffected by TSH and the combination with ghrelin.

## Discussion

The present study is the first to investigate whether ghrelin acts directly on human thyrocytes, that have previously been described to express the GhrR [4, 55, 56], and accordingly the ability to modulate the secretion of several key components that are important to the production of thyroid hormones.

We confirmed the presence of GhrR on the thyroid tissue as well as on the cultured thyrocytes. The expression levels were lower in the cultured thyrocytes compared to the tissue samples but the expression levels were stable from day 5–6 to day 10–13 of culture (Fig 1). We showed that ghrelin decreased the TSH-induced protein level of Tg significantly for the lowest concentration of TSH and only a trend was observed for the two higher concentrations. The non-significance for these two concentrations might have been due to a large inter-culture variation of 19.9% [53] combined with the lower number of cultures at these two concentrations. Additionally, the mRNA expression of TPO was decreased and a tendency of inhibition of NIS, Tg and TSH-R was observed in thyrocytes from paranodular thyroid tissue, indicating a suppressive role of ghrelin on the thyrocytes (Figs 2 and 3). The production of Tg is influenced by the amount of TPO and NIS, hence ghrelin may mediate its inhibitory effect on Tg through NIS and/or TPO. Importantly, ghrelin did not affect the basal level of Tg and cAMP (S2 Fig), NIS, TPO or TSH-R in the absence of TSH. To explore where in the TSH signaling pathway ghrelin could cause its influence, we measured the concentration of cAMP in the cultures with or without ghrelin. This particular component is relevant because the TSH receptor is G_αs_ coupled [57] and therefore induces cAMP expression when activated in contrast to the GhrR which is G_αq_ coupled [58, 59]. We found no change in cAMP production when ghrelin was added along with TSH, suggesting that ghrelin influences the TSH pathway downstream of the adenylate cyclase (Fig 2B).

Therefore, our results indicate that there could be an antagonizing function of ghrelin on the TSH-induced function of human thyrocytes; although a direct translation from our *ex vivo* experiments to the real situation should be made with caution. Importantly, a limit of our study is that it does not take into account the feed-back mechanisms which occur in a whole organism.

### Similar studies in rats

An effect of ghrelin downstream of the adenylate cyclase is apparently supported by an *in vitro* study of a rat cell line (FRTL-5) which found evidence of crosstalk occurring downstream of cAMP through ghrelin-induced intracellular calcium signaling which changed the TSH-induced proliferation of the thyrocytes, possibly mediated by the p66Shc pathway [48]. This led to an enhanced proliferation of the thyrocytes, whereas the function of the cells remained unexamined. Another study in rat tumor thyroid cells (PC-CI3) found a potentiation of the TSH-induced expression of Tg, TPO and NIS by ghrelin [49]. However, cell lines are known to lose properties by passaging and therefore are not necessarily good markers of human physiology [60, 61]. Furthermore, studies have shown that thyroid cell lines, as a result of dedifferentiation during passaging, become highly proliferative, but often lack their primary function e.g. producing Tg, wherefore proliferation rate and function do not consistently correlate positively [61]. Our study is the only one investigating the effect of ghrelin on *human* thyroid tissue and moreover in *primary* cell cultures.

### New contributions to understand the role of ghrelin on the hypothalamus-pituitary-thyroid-axis

Thus, our findings are new contributions to understanding the complex effects of ghrelin on the HPT axis and thereby on the levels of thyroid hormones in health and disease. It may therefore contribute to our understanding of the correlation between the plasma levels of ghrelin and thyroid hormones. The inhibiting effect of ghrelin on the thyroid components found in this study could be due to an energy saving strategy in which the orexigenic effect of ghrelin together with the decreased metabolism leads to a less catabolic state. This is in accordance with the inverse relationship between thyroid hormones and ghrelin found in patients with especially hyperthyroidism [18–29] as well as the inhibiting effect of ghrelin on the HPT axis shown in several *in vivo* studies [5, 34–40]. The adipogenic effect of ghrelin has until now mostly been attributed to increased food intake and increased fat accumulation but with this study we propose a new mechanism, though, more studies need to be done to clarify mechanisms.

## Conclusions

This study demonstrates for the first time a direct effect of ghrelin on human thyrocytes *ex vivo* and thereby suggests a new possible role for ghrelin in regulating the production of thyroid hormones in humans. The shown suppressive impact of ghrelin on the thyrocytes is one more minor step towards understanding the role of ghrelin in human energy homeostasis and may in the long run contribute to the development of new therapeutic strategies in thyroid and metabolic disorders.

## Supporting information

S1 Fig The influence of ghrelin on the thyroid-stimulating hormone (TSH)-induced increase in thyroglobulin (Tg) and cAMP production for each patient. The influence of ghrelin on the TSH-induced increase in thyroglobulin Tg and cAMP production at three different concentrations of TSH (0.1 IU/L, 0.5 IU/L and 1 IU/L). The basal levels, i.e. the values in the absence of TSH, were subtracted, before the groups were compared. Grey = vehicle, pattern = ghrelin (100 nM). Means (+SEM). *P < 0.05 compared to the control (vehicle). **A)** Ghrelin inhibited the TSH-induced increase in Tg production measured by enzyme-linked immunosorbent assay (ELISA) in primary cultures of human thyroid cells for the TSH concentration of 0.1 IU/L. n = 8 (0.1 IU/L) and n = 6 (0.5 and 1 IU/L) in triplets. Two patient samples were excluded due to lack of basal TSH-induced Tg production. **B)** No influence of ghrelin on the TSH-induced increase in cAMP production at three different concentrations of TSH (0.1 IU/L, 0.5 IU/L and 1 IU/L) measured by a competitive protein binding method in primary cultures of human thyroid cells. n = 8 (0.1 IU/L and 1 IU/L) and n = 6 (0.5 IU/L) in triplets.
(TIF)

S2 Fig The influence of ghrelin on the level of thyroglobulin (Tg) and cAMP in the absence of TSH. Grey = vehicle (without ghrelin), pattern = ghrelin (100 nM). Means (+SEM). *P < 0.05 compared to the control (vehicle). n = 8 in triplets. **A)** Ghrelin did not influence the basal level of Tg (μg/L) in the absence of TSH measured by enzyme-linked immunosorbent assay (ELISA) in primary cultures of human thyroid cells. **B)** No influence of ghrelin on the basal level of cAMP (μmol/L) was observed in the absence of TSH, when measured using a competitive protein binding method in primary cultures of human thyroid cells.
(TIF)

## References

[1] Sivertsen B, Holliday N, Madsen AN, Holst B. Functionally biased signalling properties of 7TM receptors—opportunities for drug development for the ghrelin receptor. British journal of pharmacology. 2013;170(7):1349–62. Epub 2013/09/17. doi: 10.1111/bph.12361 ; PubMed Central PMCID: PMCPMC3838681. 24032557 10.1111/bph.12361 PMC3838681

[2] Howard AD, Feighner SD, Cully DF, Arena JP, Liberator PA, Rosenblum CI, et al A receptor in pituitary and hypothalamus that functions in growth hormone release. Science (New York, NY). 1996;273(5277):974–7. Epub 1996/08/16. . 8688086 10.1126/science.273.5277.974

[3] Zigman JM, Jones JE, Lee CE, Saper CB, Elmquist JK. Expression of ghrelin receptor mRNA in the rat and the mouse brain. The Journal of comparative neurology. 2006;494(3):528–48. Epub 2005/12/02. doi: 10.1002/cne.20823 ; PubMed Central PMCID: PMCPMC4524499. 16320257 10.1002/cne.20823 PMC4524499

[4] Gnanapavan S, Kola B, Bustin SA, Morris DG, McGee P, Fairclough P, et al The tissue distribution of the mRNA of ghrelin and subtypes of its receptor, GHS-R, in humans. The Journal of clinical endocrinology and metabolism. 2002;87(6):2988 Epub 2002/06/07. doi: 10.1210/jcem.87.6.8739 . 12050285 10.1210/jcem.87.6.8739

[5] Wren AM, Small CJ, Ward HL, Murphy KG, Dakin CL, Taheri S, et al The novel hypothalamic peptide ghrelin stimulates food intake and growth hormone secretion. Endocrinology. 2000;141(11):4325–8. Epub 2000/11/23. doi: 10.1210/endo.141.11.7873 . 11089570 10.1210/endo.141.11.7873

[6] Tang-Christensen M, Vrang N, Ortmann S, Bidlingmaier M, Horvath TL, Tschop M. Central administration of ghrelin and agouti-related protein (83–132) increases food intake and decreases spontaneous locomotor activity in rats. Endocrinology. 2004;145(10):4645–52. Epub 2004/07/03. doi: 10.1210/en.2004-0529 . 15231700 10.1210/en.2004-0529

[7] Theander-Carrillo C, Wiedmer P, Cettour-Rose P, Nogueiras R, Perez-Tilve D, Pfluger P, et al Ghrelin action in the brain controls adipocyte metabolism. The Journal of clinical investigation. 2006;116(7):1983–93. Epub 2006/06/13. doi: 10.1172/JCI25811 ; PubMed Central PMCID: PMCPMC1474815. 16767221 10.1172/JCI25811 PMC1474815

[8] Tschop M, Smiley DL, Heiman ML. Ghrelin induces adiposity in rodents. Nature. 2000;407(6806):908–13. Epub 2000/11/01. doi: 10.1038/35038090 . 11057670 10.1038/35038090

[9] Marzullo P, Verti B, Savia G, Walker GE, Guzzaloni G, Tagliaferri M, et al The relationship between active ghrelin levels and human obesity involves alterations in resting energy expenditure. The Journal of clinical endocrinology and metabolism. 2004;89(2):936–9. Epub 2004/02/07. doi: 10.1210/jc.2003-031328 . 14764817 10.1210/jc.2003-031328

[10] Kuroda K, Hequing H, Mondal A, Yoshimura M, Ito K, Mikami T, et al Ghrelin Is an Essential Factor for Motilin-Induced Gastric Contraction in Suncus murinus. Endocrinology. 2015;156(12):4437–47. Epub 2015/10/07. doi: 10.1210/en.2015-1561 . 26441238 10.1210/en.2015-1561

[11] Wang Y, Chen F, Shi H, Jiang J, Li H, Qin B, et al Extrinsic ghrelin in the paraventricular nucleus increases small intestinal motility in rats by activating central growth hormone secretagogue and enteric cholinergic receptors. Peptides. 2015;74:43–9. Epub 2015/10/04. doi: 10.1016/j.peptides.2015.09.009 . 26431788 10.1016/j.peptides.2015.09.009

[12] Haqq AM, Farooqi IS, O'Rahilly S, Stadler DD, Rosenfeld RG, Pratt KL, et al Serum ghrelin levels are inversely correlated with body mass index, age, and insulin concentrations in normal children and are markedly increased in Prader-Willi syndrome. The Journal of clinical endocrinology and metabolism. 2003;88(1):174–8. Epub 2003/01/10. doi: 10.1210/jc.2002-021052 . 12519848 10.1210/jc.2002-021052

[13] Tschop M, Weyer C, Tataranni PA, Devanarayan V, Ravussin E, Heiman ML. Circulating ghrelin levels are decreased in human obesity. Diabetes. 2001;50(4):707–9. Epub 2001/04/06. . 11289032 10.2337/diabetes.50.4.707

[14] Cummings DE, Weigle DS, Frayo RS, Breen PA, Ma MK, Dellinger EP, et al Plasma ghrelin levels after diet-induced weight loss or gastric bypass surgery. The New England journal of medicine. 2002;346(21):1623–30. Epub 2002/05/25. doi: 10.1056/NEJMoa012908 . 12023994 10.1056/NEJMoa012908

[15] Otto B, Cuntz U, Fruehauf E, Wawarta R, Folwaczny C, Riepl RL, et al Weight gain decreases elevated plasma ghrelin concentrations of patients with anorexia nervosa. European journal of endocrinology / European Federation of Endocrine Societies. 2001;145(5):669–73. Epub 2001/11/27. . 11720888

[16] Zhu Y, Hsu WH, Hollis JH. Modified sham feeding of foods with different macronutrient compositions differentially influences cephalic change of insulin, ghrelin, and NMR-based metabolomic profiles. Physiology & behavior. 2014;135:135–42. Epub 2014/06/22. doi: 10.1016/j.physbeh.2014.06.009 . 24952264 10.1016/j.physbeh.2014.06.009

[17] Schussler P, Kluge M, Yassouridis A, Dresler M, Uhr M, Steiger A. Ghrelin levels increase after pictures showing food. Obesity (Silver Spring, Md). 2012;20(6):1212–7. Epub 2012/01/14. doi: 10.1038/oby.2011.385 . 22240720 10.1038/oby.2011.385

[18] Ruchala M, Gurgul E, Stangierski A, Wrotkowska E, Moczko J. Individual plasma ghrelin changes in the same patients in hyperthyroid, hypothyroid and euthyroid state. Peptides. 2014;51:31–4. Epub 2013/11/05. doi: 10.1016/j.peptides.2013.10.018 . 24184592 10.1016/j.peptides.2013.10.018

[19] Gurgul E, Ruchala M, Kosowicz J, Zamyslowska H, Wrotkowska E, Moczko J, et al Ghrelin and obestatin in thyroid dysfunction. Endokrynologia Polska. 2012;63(6):456–62. Epub 2013/01/23. . 23339003

[20] Kosowicz J, Baumann-Antczak A, Ruchala M, Gryczynska M, Gurgul E, Sowinski J. Thyroid hormones affect plasma ghrelin and obestatin levels. Hormone and metabolic research = Hormon- und Stoffwechselforschung = Hormones et metabolisme. 2011;43(2):121–5. Epub 2010/12/18. doi: 10.1055/s-0030-1269853 . 21165813 10.1055/s-0030-1269853

[21] Gjedde S, Vestergaard ET, Gormsen LC, Riis AL, Rungby J, Moller N, et al Serum ghrelin levels are increased in hypothyroid patients and become normalized by L-thyroxine treatment. The Journal of clinical endocrinology and metabolism. 2008;93(6):2277–80. Epub 2008/04/03. doi: 10.1210/jc.2007-2619 . 18381578 10.1210/jc.2007-2619

[22] Braclik M, Marcisz C, Giebel S, Orzel A. Serum leptin and ghrelin levels in premenopausal women with stable body mass index during treatment of thyroid dysfunction. Thyroid: official journal of the American Thyroid Association. 2008;18(5):545–50. Epub 2008/05/10. doi: 10.1089/thy.2007.0300 . 18466078 10.1089/thy.2007.0300

[23] Kokkinos A, Mourouzis I, Kyriaki D, Pantos C, Katsilambros N, Cokkinos DV. Possible implications of leptin, adiponectin and ghrelin in the regulation of energy homeostasis by thyroid hormone. Endocrine. 2007;32(1):30–2. Epub 2007/11/10. doi: 10.1007/s12020-007-9002-5 . 17992599 10.1007/s12020-007-9002-5

[24] Tanda ML, Lombardi V, Genovesi M, Ultimieri F, Lai A, Gandolfo M, et al Plasma total and acylated Ghrelin concentrations in patients with clinical and subclinical thyroid dysfunction. Journal of endocrinological investigation. 2009;32(1):74–8. . 19337020 10.1007/BF03345683

[25] Gimenez-Palop O, Gimenez-Perez G, Mauricio D, Berlanga E, Potau N, Vilardell C, et al Circulating ghrelin in thyroid dysfunction is related to insulin resistance and not to hunger, food intake or anthropometric changes. European journal of endocrinology / European Federation of Endocrine Societies. 2005;153(1):73–9. Epub 2005/07/05. doi: 10.1530/eje.1.01934 . 15994748 10.1530/eje.1.01934

[26] Sawicka B, Bossowski A, Szalecki M, Wysoka J, Koput A, Zelazowska-Rutkowska B, et al Relationship between metabolic parameters and thyroid hormones and the level of gastric peptides in children with autoimmune thyroid diseases. Journal of pediatric endocrinology & metabolism: JPEM. 2010;23(4):345–54. Epub 2010/06/30. . 20583539 10.1515/jpem.2010.055

[27] El Gawad SS, El Kenawy F, Mousa AA, Omar AA. Plasma levels of resistin and ghrelin before and after treatment in patients with hyperthyroidism. Endocrine practice: official journal of the American College of Endocrinology and the American Association of Clinical Endocrinologists. 2012;18(3):376–81. Epub 2011/07/12. doi: 10.4158/ep11130.or . 21742599 10.4158/EP11130.OR

[28] Sawicka B, Bossowski A, Urban M, Szalecki M, Wysocka J, Koput A, et al [Analysis of serum levels of ghrelin and obestatin in children and adolescents with autoimmune thyroid diseases]. Pediatric endocrinology, diabetes, and metabolism. 2009;15(1):20–7. Epub 2009/05/21. . 19454185

[29] Riis AL, Hansen TK, Moller N, Weeke J, Jorgensen JO. Hyperthyroidism is associated with suppressed circulating ghrelin levels. The Journal of clinical endocrinology and metabolism. 2003;88(2):853–7. Epub 2003/02/08. doi: 10.1210/jc.2002-021302 . 12574224 10.1210/jc.2002-021302

[30] Malandrino N, Miceli A, Leggio L, Mingrone G, Capristo E. High ghrelin levels in post-treatment euthyroid patients with Hashimoto's thyroiditis: a case-control preliminary study. Experimental and clinical endocrinology & diabetes: official journal, German Society of Endocrinology [and] German Diabetes Association. 2014;122(9):540–3. Epub 2014/06/12. doi: 10.1055/s-0034-1376965 . 24918532 10.1055/s-0034-1376965 PMC12440139

[31] Altinova AE, Toruner F, Karakoc A, Yetkin I, Ayvaz G, Cakir N, et al Serum Ghrelin Levels in patients with Hashimoto's thyroiditis. Thyroid: official journal of the American Thyroid Association. 2006;16(12):1259–64. Epub 2007/01/04. doi: 10.1089/thy.2006.16.1259 . 17199436 10.1089/thy.2006.16.1259

[32] Biyikli HH, Arduc A, Isik S, Ozuguz U, Caner S, Dogru F, et al Assessing the Relationship Between Serum Ghrelin Levels and Metabolic Parameters and Autoimmunity in Patients with Euthyroid Hashimoto's Thyroiditis. Endocrine practice: official journal of the American College of Endocrinology and the American Association of Clinical Endocrinologists. 2014;20(8):818–24. Epub 2014/02/13. doi: 10.4158/ep13469.or . 24518184 10.4158/EP13469.OR

[33] Emami A, Nazem R, Hedayati M. Is association between thyroid hormones and gut peptides, ghrelin and obestatin, able to suggest new regulatory relation between the HPT axis and gut? Regulatory peptides. 2014;189:17–21. Epub 2014/02/11. doi: 10.1016/j.regpep.2014.01.001 . 24508278 10.1016/j.regpep.2014.01.001

[34] Sosic-Jurjevic B, Stevanovic D, Milosevic V, Sekulic M, Starcevic V. Central ghrelin affects pituitary-thyroid axis: histomorphological and hormonal study in rats. Neuroendocrinology. 2009;89(3):327–36. Epub 2009/01/06. doi: 10.1159/000188603 . 19122448 10.1159/000188603

[35] Pekary AE, Sattin A. Rapid modulation of TRH and TRH-like peptide release in rat brain and peripheral tissues by ghrelin and 3-TRP-ghrelin. Peptides. 2012;36(2):157–67. Epub 2012/05/29. doi: 10.1016/j.peptides.2012.04.021 . 22634385 10.1016/j.peptides.2012.04.021

[36] Kluge M, Riedl S, Uhr M, Schmidt D, Zhang X, Yassouridis A, et al Ghrelin affects the hypothalamus-pituitary-thyroid axis in humans by increasing free thyroxine and decreasing TSH in plasma. Eur J Endocrinol. 2010;162(6):1059–65. Epub 2010/04/29. doi: 10.1530/EJE-10-0094 . 20423986 10.1530/EJE-10-0094

[37] Fusco A, Bianchi A, Mancini A, Milardi D, Giampietro A, Cimino V, et al Effects of ghrelin administration on endocrine and metabolic parameters in obese women with polycystic ovary syndrome. Journal of endocrinological investigation. 2007;30(11):948–56. Epub 2008/02/06. doi: 10.1007/BF03349243 . 18250617 10.1007/BF03349243

[38] Kluge M, Schmidt D, Uhr M, Steiger A. Ghrelin suppresses nocturnal secretion of luteinizing hormone (LH) and thyroid stimulating hormone (TSH) in patients with major depression. Journal of psychiatric research. 2013;47(9):1236–9. Epub 2013/06/04. doi: 10.1016/j.jpsychires.2013.05.010 . 23726373 10.1016/j.jpsychires.2013.05.010

[39] Kordi F, Khazali H. The effect of ghrelin and estradiol on mean concentration of thyroid hormones. International journal of endocrinology and metabolism. 2015;13(1):e17988 Epub 2015/03/10. doi: 10.5812/ijem.17988 ; PubMed Central PMCID: PMCPMC4338654. 25745491 10.5812/ijem.17988 PMC4338654

[40] Mahmoudi F, Mohsennezhad F, Khazali H, Ehtesham H. The effect of central injection of ghrelin and bombesin on mean plasma thyroid hormones concentration. Iranian journal of pharmaceutical research: IJPR. 2011;10(3):627–32. Epub 2011/07/01. ; PubMed Central PMCID: PMCPMC3813021. 24250396 PMC3813021

[41] Kamegai J, Tamura H, Shimizu T, Ishii S, Sugihara H, Wakabayashi I. Chronic central infusion of ghrelin increases hypothalamic neuropeptide Y and Agouti-related protein mRNA levels and body weight in rats. Diabetes. 2001;50(11):2438–43. Epub 2001/10/27. . 11679419 10.2337/diabetes.50.11.2438

[42] Fekete C, Sarkar S, Rand WM, Harney JW, Emerson CH, Bianco AC, et al Agouti-related protein (AGRP) has a central inhibitory action on the hypothalamic-pituitary-thyroid (HPT) axis; comparisons between the effect of AGRP and neuropeptide Y on energy homeostasis and the HPT axis. Endocrinology. 2002;143(10):3846–53. Epub 2002/09/20. doi: 10.1210/en.2002-220338 . 12239096 10.1210/en.2002-220338

[43] Fekete C, Marks DL, Sarkar S, Emerson CH, Rand WM, Cone RD, et al Effect of Agouti-related protein in regulation of the hypothalamic-pituitary-thyroid axis in the melanocortin 4 receptor knockout mouse. Endocrinology. 2004;145(11):4816–21. Epub 2004/07/17. doi: 10.1210/en.2004-0476 . 15256492 10.1210/en.2004-0476

[44] Fekete C, Kelly J, Mihaly E, Sarkar S, Rand WM, Legradi G, et al Neuropeptide Y has a central inhibitory action on the hypothalamic-pituitary-thyroid axis. Endocrinology. 2001;142(6):2606–13. Epub 2001/05/18. doi: 10.1210/endo.142.6.8207 . 11356711 10.1210/endo.142.6.8207

[45] Fekete C, Singru PS, Sanchez E, Sarkar S, Christoffolete MA, Riberio RS, et al Differential effects of central leptin, insulin, or glucose administration during fasting on the hypothalamic-pituitary-thyroid axis and feeding-related neurons in the arcuate nucleus. Endocrinology. 2006;147(1):520–9. Epub 2005/10/08. doi: 10.1210/en.2005-0956 . 16210367 10.1210/en.2005-0956

[46] Pazos Y, Casanueva FF, Camina JP. Basic aspects of ghrelin action. Vitamins and hormones. 2008;77:89–119. Epub 2007/11/07. doi: 10.1016/S0083-6729(06)77005-4 . 17983854 10.1016/S0083-6729(06)77005-4

[47] Reichenbach A, Steyn FJ, Sleeman MW, Andrews ZB. Ghrelin receptor expression and colocalization with anterior pituitary hormones using a GHSR-GFP mouse line. Endocrinology. 2012;153(11):5452–66. Epub 2012/09/11. doi: 10.1210/en.2012-1622 . 22962259 10.1210/en.2012-1622

[48] Park YJ, Lee YJ, Kim SH, Joung DS, Kim BJ, So I, et al Ghrelin enhances the proliferating effect of thyroid stimulating hormone in FRTL-5 thyroid cells. Molecular and cellular endocrinology. 2008;285(1–2):19–25. Epub 2008/03/04. doi: 10.1016/j.mce.2008.01.003 . 18313206 10.1016/j.mce.2008.01.003

[49] Morillo-Bernal J, Fernandez-Santos JM, De Miguel M, Garcia-Marin R, Gordillo-Martinez F, Diaz-Parrado E, et al Ghrelin potentiates TSH-induced expression of the thyroid tissue-specific genes thyroglobulin, thyroperoxidase and sodium-iodine symporter, in rat PC-Cl3 Cells. Peptides. 2011;32(11):2333–9. Epub 2011/09/29. doi: 10.1016/j.peptides.2011.09.013 . 21945915 10.1016/j.peptides.2011.09.013

[50] Ambesi-Impiombato FS, Parks LA, Coon HG. Culture of hormone-dependent functional epithelial cells from rat thyroids. Proceedings of the National Academy of Sciences of the United States of America. 1980;77(6):3455–9. Epub 1980/06/01. ; PubMed Central PMCID: PMCPMC349635. 6106191 10.1073/pnas.77.6.3455 PMC349635

[51] Madsen SN, Badawi I, Skovsted L. A simple competitive protein-binding assay for adenosine-3',5'-monophosphate in plasma and urine. Acta endocrinologica. 1976;81(1):208–14. Epub 1976/01/01. . 174364 10.1530/acta.0.0810208

[52] Kronborg TM, Hansen JF, Rasmussen AK, Vorkamp K, Nielsen CH, Frederiksen M, et al The flame retardant DE-71 (a mixture of polybrominated diphenyl ethers) inhibits human differentiated thyroid cell function in vitro. PloS one. 2017;12(6):e0179858 Epub 2017/06/24. doi: 10.1371/journal.pone.0179858 ; PubMed Central PMCID: PMCPMC5482471. 28644858 10.1371/journal.pone.0179858 PMC5482471

[53] Rasmussen AK, Kayser L, Perrild H, Brandt M, Bech K, Feldt-Rasmussen U. Human thyroid epithelial cells cultured in monolayers. II. Influence of serum on thyroglobulin and cAMP production. Molecular and cellular endocrinology. 1996;116(2):173–9. Epub 1996/02/05. . 8647317 10.1016/0303-7207(95)03712-8

[54] Roger P, Taton M, Van Sande J, Dumont JE. Mitogenic effects of thyrotropin and adenosine 3',5'-monophosphate in differentiated normal human thyroid cells in vitro. The Journal of clinical endocrinology and metabolism. 1988;66(6):1158–65. Epub 1988/06/01. doi: 10.1210/jcem-66-6-1158 . 2836470 10.1210/jcem-66-6-1158

[55] Cassoni P, Papotti M, Catapano F, Ghe C, Deghenghi R, Ghigo E, et al Specific binding sites for synthetic growth hormone secretagogues in non-tumoral and neoplastic human thyroid tissue. The Journal of endocrinology. 2000;165(1):139–46. Epub 2000/04/06. . 10750044 10.1677/joe.0.1650139

[56] Konturek PC, Brzozowski T, Pajdo R, Nikiforuk A, Kwiecien S, Harsch I, et al Ghrelin-a new gastroprotective factor in gastric mucosa. Journal of physiology and pharmacology: an official journal of the Polish Physiological Society. 2004;55(2):325–36. Epub 2004/06/24. . 15213356

[57] Calebiro D, Nikolaev VO, Lohse MJ. Imaging of persistent cAMP signaling by internalized G protein-coupled receptors. Journal of molecular endocrinology. 2010;45(1):1–8. Epub 2010/04/10. doi: 10.1677/JME-10-0014 . 20378719 10.1677/JME-10-0014

[58] Holst B, Cygankiewicz A, Jensen TH, Ankersen M, Schwartz TW. High constitutive signaling of the ghrelin receptor—identification of a potent inverse agonist. Molecular endocrinology (Baltimore, Md). 2003;17(11):2201–10. Epub 2003/08/09. doi: 10.1210/me.2003-0069 . 12907757 10.1210/me.2003-0069

[59] Yin Y, Li Y, Zhang W. The growth hormone secretagogue receptor: its intracellular signaling and regulation. International journal of molecular sciences. 2014;15(3):4837–55. Epub 2014/03/22. doi: 10.3390/ijms15034837 ; PubMed Central PMCID: PMCPMC3975427. 24651458 10.3390/ijms15034837 PMC3975427

[60] Bellur S, Tahara K, Saji M, Grollman EF, Kohn LD. Repeatedly passed FRTL-5 rat thyroid cells can develop insulin and insulin-like growth factor-I-sensitive cyclooxygenase and prostaglandin E2 isomerase-like activities together with altered basal and thyrotropin-responsive thymidine incorporation into DNA. Endocrinology. 1990;127(3):1526–40. Epub 1990/09/01. doi: 10.1210/endo-127-3-1526 . 2167218 10.1210/endo-127-3-1526

[61] Zimmermann-Belsing T, Rasmussen AK, Feldt-Rasmussen U. Lack of thyroglobulin synthesis as an indicator of early random dedifferentiation of the Fischer rat thyroid cell line FRTL-5. Scandinavian journal of clinical and laboratory investigation. 1998;58(7):529–35. Epub 1999/01/16. . 9890335 10.1080/00365519850186148

