# Supplementary figures and images for: Ghrelin-mediated inhibition of the TSH-stimulated function of differentiated human thyrocytes ex vivo
Source: PLoS One. 2017 Sep 20;12(9):e0184992. doi: 10.1371/journal.pone.0184992 (PMC5607171; doi:10.1371/journal.pone.0184992)

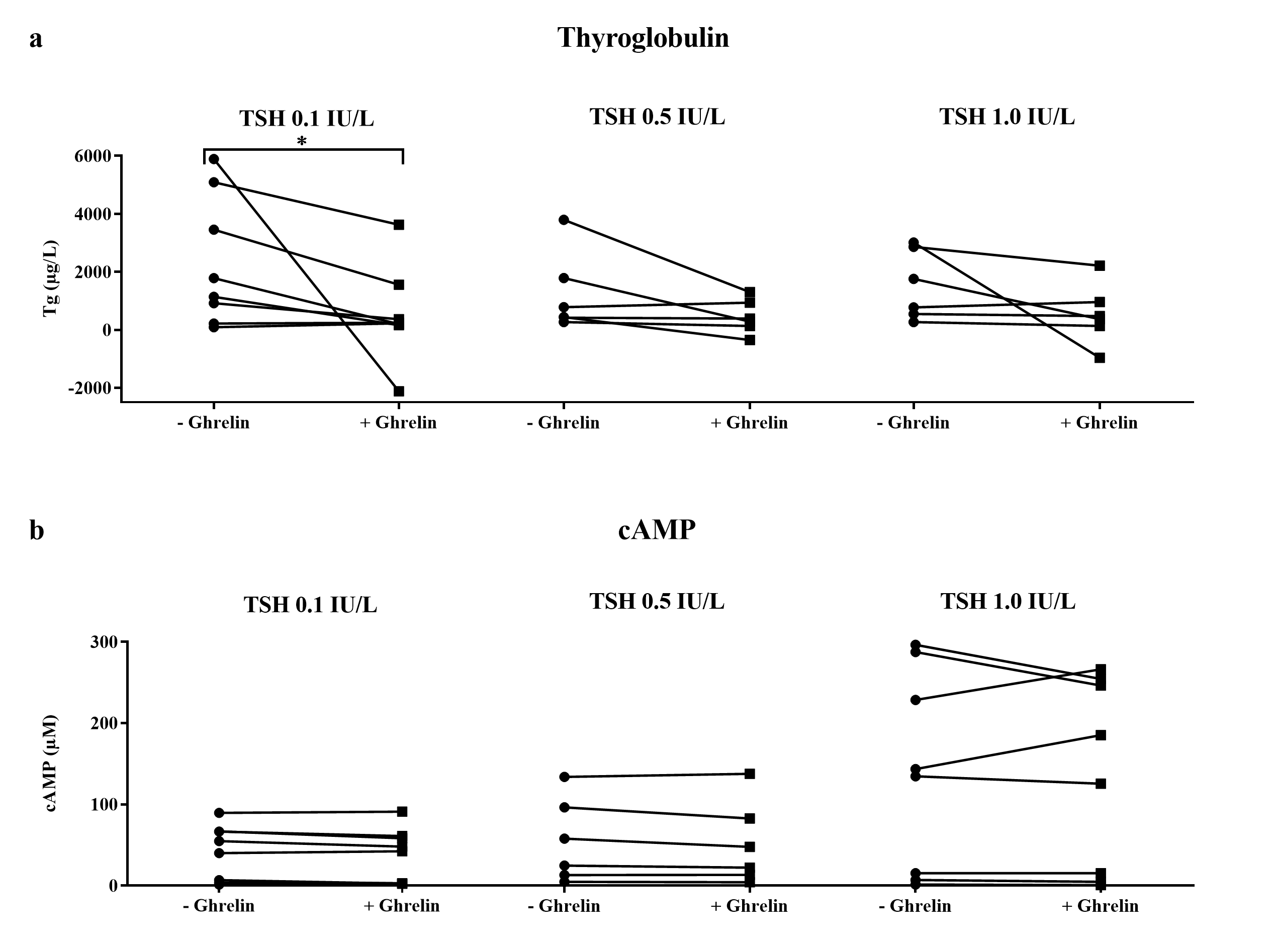

Supplement: S1 Fig — The influence of ghrelin on the TSH-induced increase in thyroglobulin Tg and cAMP production at three different concentrations of TSH (0.1 IU/L, 0.5 IU/L and 1 IU/L). The basal levels, i.e. the values in the absence of TSH, were subtracted, before the groups were compared. Grey = vehicle, pattern = ghrelin (100 nM). Means (+SEM). *P < 0.05 compared to the control (vehicle). A) Ghrelin inhibited the TSH-induced increase in Tg production measured by enzyme-linked immunosorbent assay (ELISA) in primary cultures of human thyroid cells for the TSH concentration of 0.1 IU/L. n = 8 (0.1 IU/L) and n = 6 (0.5 and 1 IU/L) in triplets. Two patient samples were excluded due to lack of basal TSH-induced Tg production. B) No influence of ghrelin on the TSH-induced increase in cAMP production at three different concentrations of TSH (0.1 IU/L, 0.5 IU/L and 1 IU/L) measured by a competitive protein binding method in primary cultures of human thyroid cells. n = 8 (0.1 IU/L and 1 IU/L) and n = 6 (0.5 IU/L) in triplets. (TIF) [file pone.0184992.s001.tif]

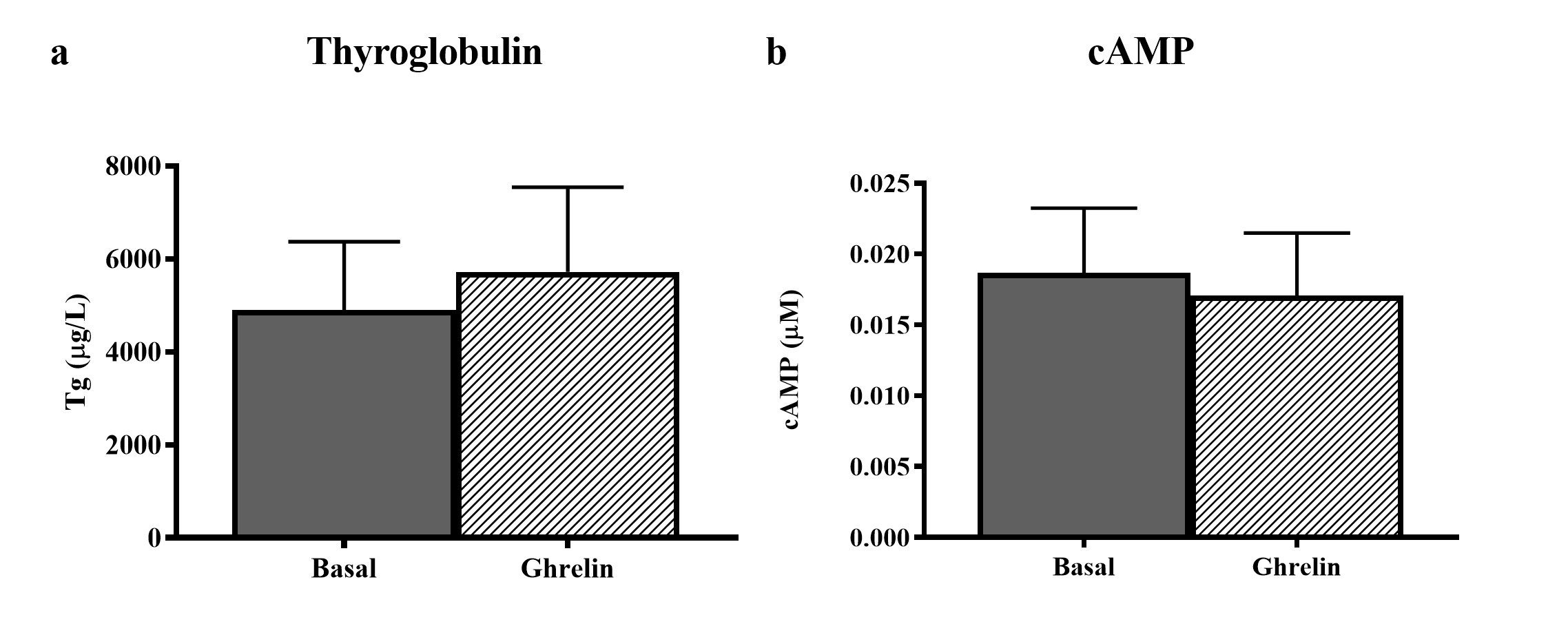

Supplement: S2 Fig — Grey = vehicle (without ghrelin), pattern = ghrelin (100 nM). Means (+SEM). *P < 0.05 compared to the control (vehicle). n = 8 in triplets. A) Ghrelin did not influence the basal level of Tg (μg/L) in the absence of TSH measured by enzyme-linked immunosorbent assay (ELISA) in primary cultures of human thyroid cells. B) No influence of ghrelin on the basal level of cAMP (μmol/L) was observed in the absence of TSH, when measured using a competitive protein binding method in primary cultures of human thyroid cells. (TIF) [file pone.0184992.s002.tif]
